# Characterization of the In Vitro Cultured Ovarian Cells in the Asian Yellow Pond Turtle (*Mauremys mutica*)

**DOI:** 10.3390/biology11101404

**Published:** 2022-09-26

**Authors:** Xiaoli Liu, Fang Liu, Haoyang Xu, Yanping Yang, Yakun Wang, Xiaoyou Hong, Wei Li, Lingyun Yu, Chen Chen, Hongyan Xu, Xinping Zhu

**Affiliations:** 1 Key Laboratory of Tropical and Subtropical Fishery Resources Application and Cultivation, Ministry of Agriculture and Rural Affairs, Pearl River Fisheries Research Institute, Chinese Academy of Fishery Sciences, Guangzhou 510380, China; 2 Key Laboratory of Freshwater Fish Reproduction and Development (Ministry of Education), College of Fisheries, Southwest University, Chongqing 402460, China

**Keywords:** Asian yellow pond turtle, female germline stem cells (fGSCs), germ cells, ovarian cells, cell development

## Abstract

**Simple Summary:**

Gonadal cell lines possess the abilities of self-renewal and differentiation, being used as an efficient tool to analyzing the genes’ functions involved in sex differentiation and gametogenesis. Although some significant achievements have been obtained in the gonadal cells’ culture or manipulation across multiple phyla including teleost and mammals, there is limited study on gonadal cell manipulation in turtles. In this study, we isolated an ovarian cell line (fGSCs), from the juvenile Asian yellow pond turtle in vitro. The cultured fGSCs were characterized with germline stem cell specific markers’ expression using transcriptome sequencing on Illumina platform, reverse transcription-polymerase chain reaction (RT-PCR) and immunofluorescence (IF). Our study aims to provide a valuable tool to elaborate the molecular mechanisms behind germ cells development, differentiation and oogenesis in the turtle even in reptiles, and also, the established cell line has potential applications to enrich the conservation strategy of aquatic animal germplasm resources.

**Abstract:**

Gonadal cell lines possess the abilities of self-renewal and differentiation, being used as an efficient tool to analyzing the genes’ functions involved in sex differentiation and gametogenesis. Although some significant achievements have been obtained in the gonadal cells’ culture or manipulation across multiple phyla including teleost and mammals, there is limited study on gonadal cell manipulation in turtles. In this study, we established a new ovarian cell line from the young Asian yellow pond turtle (*Mauremys mutica*), which exhibited a normal diploid karyotype with high alkaline phosphatase activity. The cell line, designated as YTO2, was then characterized through the analysis of gene expression profiles. The transcriptome analysis and the reverse transcription polymerase chain reaction (RT-PCR) showed that the cells expressed germline genes such as *tdrd7*, *nanos1*, *klf5*, *igtb1*, *hsd17b4* and *rad51*. Moreover, the immunostaining showed that the germ cell markers, Tdrd7 and Rad51 proteins, were detected predominant in cytoplasm of perinuclear region, while proliferation marker, PCNA, was dominantly observed in the nuclei of cultured cells. Intriguingly, the cells could respond to the retinoic acid induction with significantly increasing the expression level of some meiosis genes, including *vasa*, *dazl*, *figla*, and *dmc1*. Furthermore, YTO2 cells could be efficiently transfected with the pHBAd-BHG-EGFP adenovirus and properly expressed the exogenous genes. To sum up, an ovarian cell line of the Asian yellow pond turtle had been established and could be stably propagated under in vitro culture condition, as well as being capable of efficiently expressing the exogenous gene *tdrd7*. This cell line would provide a valuable tool to elaborate the molecular mechanisms behind germ cells development, differentiation and oogenesis in the turtle, even in reptiles.

## 1. Introduction

Germline stem cells (GSCs) are the founder cell population committed to producing gametes and transmit genetic information to the next generation. In male mammals, the continuous self-renewal cell population now known as spermatogonial stem cells (SSCs) are capable of producing sperm throughout its entire lifetime [1]. In the female, however, a long-standing dogma is that germline stem cells only exist in prenatal phase of life, and the production of ovarian oocytes ceases after birth until a line of researches reported multiple evidence and questioned this doctrine [2,3,4,5,6]. For the past few years, the female germline stem cells (fGSCs), also known as oogonial stem cells (OSCs) have been successfully isolated and purified across phyla, from fly to humans [7,8,9,10]. The potential applications for ovarian cellular and tissue remodeling processes are numerous, particularly in veterinary and animal production fields, for example, the donor fGSCs can give rise to offspring upon being transplanted in mice and a number of other species [11]. In addition, they could also be used in clinical therapy such as infertility treatments [12], ovarian dysfunction and disease [13].

Turtles are thought to be an ancient reptile on earth based on the discovery of their earliest fossil [14]. Due to their phylogenetic status between ectotherm and endotherm vertebrates, turtles are considered as an ideal model to trace the detailed genetic evolution involved in animals’ diversity and environmental adaptation [15]. However, investigation on female germline (oogonial) stem cells is still very new and conducted in limited turtle species [16]. Moreover, over the past 30 years, numerous turtles have become highly endangered because of excessive exploitation for the pet trade or medicinal and edible purposes [17,18], together with low hatching success rate in the wild and low fertilization success rate in captivity. Therefore, studies on ovarian cell lineage of turtles would increase the understanding on the process of ovary development, and provide some new strategies for species genetic conservation.

The Asian yellow pond turtle (*Mauremys mutica*) is widely distributed in China [19], north Vietnam and Japan [20], and has become an important commercial freshwater turtle species in east and south China. The Asian yellow pond turtle has been uplisted as a critically endangered species on the International Union for the Conservation of Nature and Natural Resources (IUCN) Red List and a Class II species under key state protection in China. Moreover, in recent years, the Asian yellow pond turtle has been extensively captive-bred or farmed in China [21], “artificial reproduction” and “germ cell development” have not been applied in practice of turtle farming. Moreover, so far, CRISPR/Cas9-mediated gene editing technology has not yet been applied in turtles due to their characteristics of egg-laying breeding. 

Therefore, in the present study, we firstly isolated an ovarian cell line, designed as fGSCs, from the juvenile Asian yellow pond turtle in vitro. Furthermore, the cultured fGSCs were characterized for germline stem cell specific marker expression using reverse transcription-polymerase chain reaction (RT-PCR) and immunofluorescence (IF). In addition, the cells’ potency of expressing exogenous genes was evaluated by adenovirus-based transfection with enhanced green fluorescent protein (EGFP). Our study aims to establish a robust in vitro culture system of ovarian cells and provide a reliable tool to investigate molecular mechanisms behind oogenesis and sexual differentiation in turtles.

## 2. Materials and Methods

### 2.1. Animals and Samples

Healthy juvenile female (2 years old) Asian yellow pond turtles were collected from Guangzhou aquatic improved variety base of Pearl River Fisheries Research Institute (Guangdong province, China). All animal experiments in our research were carried out in accordance with the Guidelines of Pearl River Fisheries Research Institute, Chinese Academy of Fishery Sciences. All turtles used were treated humanely and ethically, and the experiments were approved by Laboratory Animal Ethics Committee Pearl River Fisheries Research Institute, Chinese Academy of Fishery Sciences.

### 2.2. Isolation of Cells

Telazol (tiletamine: Zolazepam = 1:1) at a rate of 25 mg/kg was administered intramuscularly to anaesthetize the Asian yellow pond turtles before sampling. The depth of anesthesia has almost been reached when there is no response to pressing the limbs, toes or tails and stimulating the cloacal mucosa. Then the dissected tissues of Asian yellow pond turtles including ovary, kidney, spleen and liver were rinsed with the sterile phosphate-buffered saline (PBS) and torn with sterile fine forceps. Then the tissue blocks were digested with 1 mg/mL collagenase I (WBC-LS004196, Worthington, USA) and 0.25% trypsin (15050065, Gibco, Shanghai, China) at room temperature as previously described [16]. The cell suspension was filtered through 40 μm cell strainers (Milipore, Billerica, MA, USA) and then centrifuged at 300× *g* for 5 min, supernatant was discarded and the cells’ pellet was re-suspended in culture cell medium and plated into the 25 cm^2^ bottles (156340, Nunc™ EasYFlask™, Thermo, Waltham, MA, USA).

### 2.3. Culture of Cells

The cells of ovary, kidney, spleen and liver were cultured with complete medium which was used for resuspending cell pellets in previous section and propagating multipotent germ line stem cells in plastic bottles (156340, Nunc™ EasYFlask™, Thermo, USA) coated with 0.2% gelatin (Sigma, cat. G1890, St. Louis, MO, USA) following the previous descriptions [16,22]. Briefly, complete medium was prepared as below, Dulbecco’s modified Eagle medium (DMEM) supplemented with 20 mmol/L Hepes, the non-protein supplement combination 6N (1 mM glutamine, 2 nM sodium selenite, 1 mM non-essential amino acids (Glycine, L-Alanine, L-Asparagine, L-Aspartic acid, L-Glutamic Acid, L-Proline and L-Serine), 1 mM sodium pyruvate, 1 mM Pen/Strep, 55 μM β-mercaptoethanol, 15% fetal bovine serum (FBS), 5 ng/mL recombinant human basic fibroblast growth factor (bFGF) (HY-001096, PeproTech, Cranbury, NJ, USA), 0.2% seabass serum and medaka embryo extracts (~0.3 embryo per mL). Asian seabass was sampled from Daya Bay Fisheries Experiment Center in Shenzhen, China. The venous blood was obtained and then the seabass serum was collected by centrifugation at 3000× *g* for 10 min at 4 °C. Medaka embryo was collected in PBS, crushed by ultrasound, the supernatant was then taken after centrifuge at 3000× *g* for 10 min at 4 °C. Cells were cultured at 28 °C and subcultured every 5–6 days in the first passages and then every 3–5 days after 10 passages, at a split ratio of 1:2 after dissociation with 0.25% trypsin (15050065, Gibco, Shanghai, China). The cells were observed and imaged under an inverted microscope equipped with Eclipse Ti2 imaging system (Nikon Corp, Tokyo, Japan).

### 2.4. Detection of Alkaline Phosphatase Activity

Cells of liver, kidney and ovary were fixed with 4% paraformaldehyde (PFA) for 10 min, washed twice with 0.2 M Tris, and then stained with 0.188 mg/mL 5-Bromo-4-Chloro-3-Indolyl Phosphate (BCIP)/0.375 mg/mL nitroblue tetrazolium chloride (NBT) substrate (#11681451001; Roche, Germany) overnight at 4 °C. After washing with PBS twice, the cells were observed and photographed under inverted microscope (Eclipse Ti2, Nikon Corp).

### 2.5. Karyotype Analysis

The cultured ovarian cells at passage 20 were used for karyotype analysis due to their uniform morphology and strong proliferation activity. Cells confluently covering the bottom of bottles were treated with 4 ug/mL Phytohemagglutinin (PHA) overnight, followed by adding 4 ug/mL colcemid for treatment at 28 °C for 2–3 h. Subsequently, the cells were dissociated with 0.25% trypsin, and the cell pellet was collected by centrifugation at 300× *g* for 5 min. Then, the cells were incubated with hypotonic solution (0.0375 M KCl) for 30 min, and fixed twice in methanol-acetic acid (3:1) for 20 min at 4 °C. The fixed cell suspension was then dropped onto clean glass slides, air-dried, and stained with 10% Giemsa solution (10 mM potassium phosphate, pH 6.8). More than 100 metaphase spreads were analyzed for each karyotype analysis.

### 2.6. RNA Preparation and Sequencing

The cultured ovarian cells at passage 20 were dissociation with 0.25% trypsin (15050065, Gibco, Shanghai, China) and collected for subsequent RNA sequencing. Two biological replicates of cultured cells were collected. Total RNA was extracted using the mirVana miRNA Isolation Kit (Ambion, Austin, TX, USA) according to the manufacturer’s protocol. RNA integrity was evaluated using the Agilent 2100 Bioanalyzer (Agilent Technologies, Santa Clara, CA, USA). The libraries were constructed using TruSeq Stranded mRNA LTSample Prep Kit (Illumina, San Diego, CA, USA) following the manufacturer’s instructions. Then these libraries were sequenced on the Illumina sequencing platform HiSeqTM 2500 and 125 bp paired-end reads were generated.

The reads containing ploy-N and the low quality reads were removed to obtain the clean reads. Then the clean reads were mapped to *M. mutica* genome using hisat2 [23]. FPKM value and count of each gene were calculated using cufflinks [24] and eXpress, respectively [25].

### 2.7. RT-PCR Analysis

The total RNAs of Asian yellow pond turtle testis, ovary, the cultured ovarian cells, liver, spleen and kidney were extracted using SV Total RNA Isolation System (Promega Corporation, Madison, WI, USA) according to the manufacturer’s instructions. RNA concentration and purity were examined using NanoDrop 2000 Spectrophotometer (Thermo Fisher Scientific, Wilmington, DE, USA). The integrity of RNA was assessed by RNA Nano 6000 Assay Kit of the Agilent Bioanalyzer 2100 System (Agilent Technologies, CA, USA) and 1.2% agarose gel electrophoresis. The isolated RNA was then transcribed to cDNA using SuperScript^TM^ IV First-Strand Synthesis System (Invitrogen, Waltham, MA, USA) following the manufacturer’s manual. The cell pluripotency and germline marker genes used in this study were referenced to previous investigations in teleost, reptiles and mammals due to limited available information about the gene expression profiles in germline stem cells and gonads of the Asian yellow pond turtle [5,16,26,27,28]. At last, the expression level of cell pluripotency makers germline marker genes *tudor domain containing 7* (*tdrd7*), DNA repair protein RAD51 homolog 1, a recombinase gene (*rad51*), *nanos C2HC-type zinc finger 1* (*nanos1*), *integrin beta1* (*igtb1*), *hydroxysteroid 17-beta dehydrogenase 4* (*hsd17b4*) and *KLF transcription factor 5* (*klf5*) were examined and characterized in the Asian yellow pond turtle based on our full-length transcription sequences (unpublished data). *β-Actin* was used as an internal control of RT-PCR. The primers for all target genes and internal control were designed and given in Table 1. For each RT-PCR reaction, the reaction volume was 50 μL with 20 ng cDNA template. PCR was run for 2 min at 94 °C, followed by 33 cycles of 94 °C for 30 s, 55–57 °C for 30 s, 72 °C for 30 s. The PCR products were separated on 2% agarose gel, imaged with a bioimaging system (Alpha Innotech, San Leandro, CA, USA) and confirmed by sequencing.

### 2.8. Exposure to Retinoic Acid (RA)

RA has been reported to induce germ cells to enter meiotic prophase by the histological detection of condensed meiotic nuclei and the expression of meiotic markers [29,30]. To further confirm the type and development stage of the cell line, the isolated and cultured ovarian cells were exposed in RA (#MB1302-1, Meilunbio, Dalian, China) which was diluted to the concentration of 30 nM, 300 nM and 1 µM with Dimethyl sulfoxide (DMSO) according to previous studies [31,32]. After 24 h, the total RNAs of the treated cells were extracted using SV Total RNA Isolation System (Promega, USA). The expression profiles of meiotic marker *figla* and dosage suppressor of mck1 homolog (*dmc1*) and germ cell markers such including *vasa* and *dazl* were examined following the method in section “RT-PCR analysis”. The DMSO (the solvent control for RA) concentration of the highest RA concentration was used to check possible effect of DMSO on the cultured ovarian cells.

### 2.9. Fluorescent Immunostaining

The polyclonal αPCNA antibody was from the previous studies [33], the polyclonal αTdrd7 and αRad51 were home-made antibodies (unpublished data). A complementary DNA (cDNA) fragment encoding amino acids 120 to 239 of *tdrd7* and 1 to –340 of *rad51* (XP_004083949.1) were inserted into the p-ET-32a + vector (EMD Biosciences (Novagen), Darmstadt, Germany) and transfected into BL21-CodonPlus (DE3) Escherichia coli cells. The bacterial cells were cultured at 37 °C overnight and induced by addition of 0.2 mM isopropyl-1-thio-β-D-galactoside (Sigma-Aldrich, Tokyo, Japan) for 4 h at 28 °C. Cells were harvested by centrifugation and disrupted by sonication, and the soluble homogenates were purified by Ni-nitrilotriacetic acid (NI-NTA) Agarose (Qiagen, Hilden, Germany) according to the manufacturer’s instructions. The recombinant protein was purified by dialyzing with PBS and used to immunize rabbits, and the antiserum was affinity-purified on antigen-coupled CNBr-activated agarose (GE Healthcare, Chicago, IL, USA).

Immunohistochemistry was performed according to the previous reports [16]. Briefly, the cells were fixed with 4% PFA for 1–2 h, followed by two sequential washes with PBS. After being blocked with 5% BSA (V900933; Sigma, Japan) in PBS for 1 h at room temperature, the cells were incubated overnight with the primary antibodies at 4 °C respectively, including αPCNA (1:500), αTdrd7 (1:100), αRad51 (1:500). The secondary antibodies for αTdrd7 and αRad51 were horseradish peroxidase (HRP) anti-rabbit IgG (BA1054; BOSTER, Wuhan, China) and, the secondary antibody for αPCNA was HRP conjugated anti-mouse IgG (BA1050, BOSTER, Wuhan, China). The next day, the cells were washed with PBST (phosphate buffered solution containing 0.1% Tween-20) and blocked with 5% goat serum in PBS for 1 h, then incubated with the secondary antibodies for 1 h at room temperature. Signals were developed using the TSA Plus Fluorescence Systems (NEL741001KT, PerkinElmer, Waltham, MA, USA). The nucleus was stained by propidium iodide (PI), and images were viewed and acquired under confocal microscope (Zesis LSM880, Oberkochen, Germany). The pre-immune serum (mouse serum and rabbit serum) was used as a negative control experiment.

### 2.10. Infection with HBAD-tdrd7-EGFP Adenovirus

The cells’ potency of expressing exogenous genes was evaluated using adenovirus (HBAD-*tdrd7*-EGFP), purchased from HanHeng Biotechnology (Shanghai) Co., Ltd. Before transfection, the cells dissociated from the 25 cm^2^ bottle (156340, Nunc™ EasYFlask™, Thermo, USA) was resuspended with 3–5 mL fresh cell complete medium. Then take a sterile centrifuge tube and add 20 μL adenovirus (1 × 10^9^ pfu) with appropriate amount of complete medium, gently mix well, and add medium till the final volume up to 100 μL. After being incubated with adenovirus-medium mixture (adenovirus: cell medium = 1:1000) at room temperature for 1 h, the infected cells were plated into 25 cm^2^ bottles (156340, Nunc™ EasYFlask™, Thermo, USA) and then cultured at 28 °C for 48–72 h. The cells were observed and photographed under an inverted fluorescence microscope (Eclipse Ti2, Nikon Corp, Japan) after virus infection.

## 3. Results

### 3.1. Morphological and Karyotypic Characterization of Ovarian Cells

**Figure 1:**
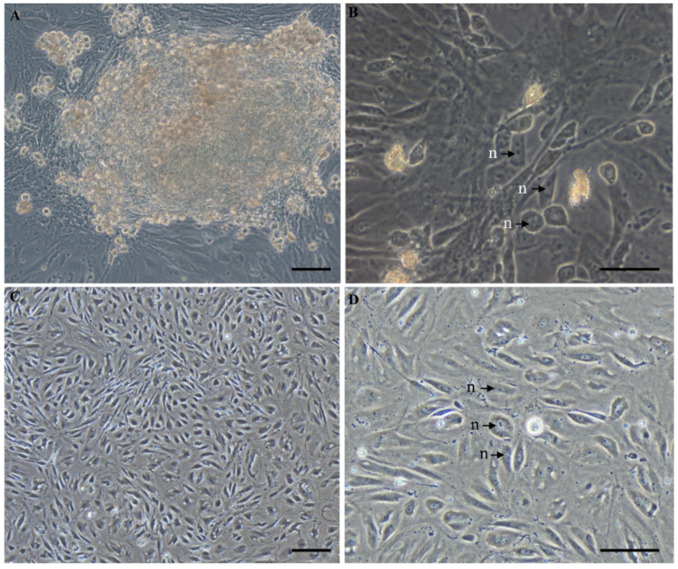
Morphology of ovarian cells during the in vitro culture. Morphological characteristics of the turtle ovarian cells line YTO2 were observed under an inverted microscope. (**A**,**B**) Ovarian cells in primary culture at Day 20. (**C**) Monolayer ovarian cells at passage 20th and Day 209th. (**D**) The cells being frozen at −196 °C for over 180 days and being thawed for culture at passage 20th and Day 209th. Arrows indicated the cells’ nucleus. Scale bar, 100 μm. “n” indicated the cells’ nuclear.

**Figure 2:**
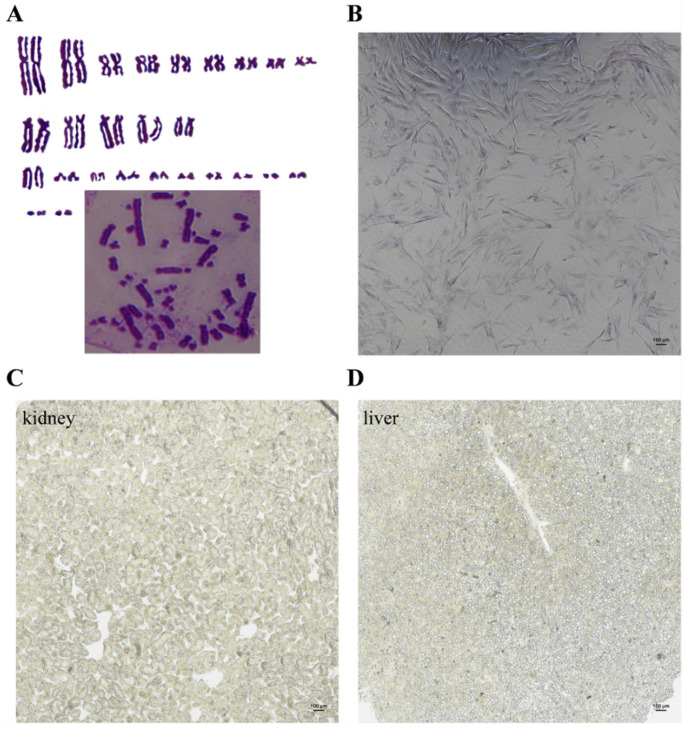
Karyotype analysis and alkaline phosphatase activity of YTO2 cells. (**A**) Karyotype analysis of the cultured ovarian cells in the Asian yellow pond turtle. Chromosomes were stained by Giemsa solution. Normal female karyotype (2n = 52) was presented in fGSCs. The lower right corner represents the original image of the chromosome. (**B**) The alkaline phosphatase activity was assessed in YTO2 cells at 20th passage. Scale bar, 100 μm. (**C**) The alkaline phosphatase activity detection of kidney cells. Scale bar, 100 μm. (**D**) The alkaline phosphatase activity detection of liver cells. Scale bar, 100 μm.

Initially, the primary cultured ovarian cells from the tissue blocks comprised a heterogeneous population with several different morphologies, such as spherically shaped, epithelial-like and fibroblast-like cells (Figure 1A,B). After 209 days of in vitro culture, from passage 20, the cells were attached to the bottom of bottle or plate and both the cell size and amount increased, thus form cell clusters with round and spindle shape colony (Figure 1C). Similar to the ovarian cells of the Chinese soft-shell turtle, the cells grew slightly during first several passages and were subcultured at intervals of 6–7 days in a ratio of 1:2. After over 10 passages, the cells started to grow fast, and should be subcultured every 3–4 days. The cells now have been successfully propagated more than 30 passages and different passages of cells were cryopreserved with the freezing medium containing 10% DMSO, 40% FBS and 50% DMEM for more than 4 years. After being thawed, the preserved cells still showed high viability and potency of proliferation similar to that before cryopreservation (Figure 1D). The morphology of passage 20 thawed cells is more uniform with approximately 8–10 μm in diameter, and most of the cells were fibroblast-like with single or multiple nuclei, while a few cells were round with 1–2 prominent nucleolus (Figure 1D). The examination of chromosome integrity showed that the ovarian cells were diploids with normal karyotype and chromosome number (2n = 52) (Figure 2A). Furthermore, most of the cells were also positive for alkaline phosphatase, which was recognized as a common stem-like cell marker (Figure 2B), while somatic cells were negative for alkaline phosphatase (Figure 2C,D). Generally, the cultured ovarian cells exhibit stable and uniform morphology, a normal diploid karyotype and high alkaline phosphatase activity, designed as YTO2.

### 3.2. Illumina Sequencing and Gene Annotation

The transcriptome analysis of cultured ovarian cells at passage 20 were performed by Illumina platform HiSeqTM 2500 to further define the characteristics of cells. Each sample of cells generated an average of 53.1 M clean reads (Table S1). An average of 93.47% and 89.26% of the clean reads were total mapped and uniquely mapped to the *M. mutica* genome (Table S1). Q30 of the two biological replicates were 94.40% and 93.85%.

**Figure 3:**
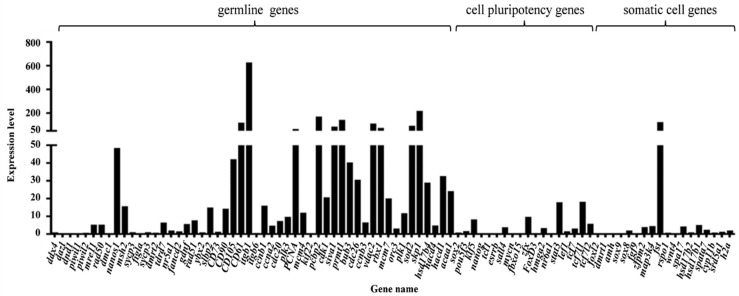
Expression level of related genes in YTO2 cells. These selected genes include well known germline marker genes, cell pluripotency makers and somatic cell genes identified.

A total of 17,216 genes were annotated to *M. mutica* genome (Table S2). Among these genes, several well known germline marker genes such as *nanos1*, *tdrd7*, *vasa*, *dnd1*, *dmc1*, cell pluripotency makers *pou5f3*, *nanog*, somatic cell genes *dmrt1*, *foxl2* as well as meiosis initiation gene, *rad51* were also identified in YTO2 (Figure 3 and Table S3). Notably, we also detected some novel genes such as *ccnb1*, *ccna2*, *cdc20*, *plk3*, *pcbp2* and *hsd17b4* (Table S3), these genes have been reported in germ cell cluster of fish and mammals [34,35,36].

### 3.3. Analysis of Marker Genes in the YTO2 cells

**Figure 4:**
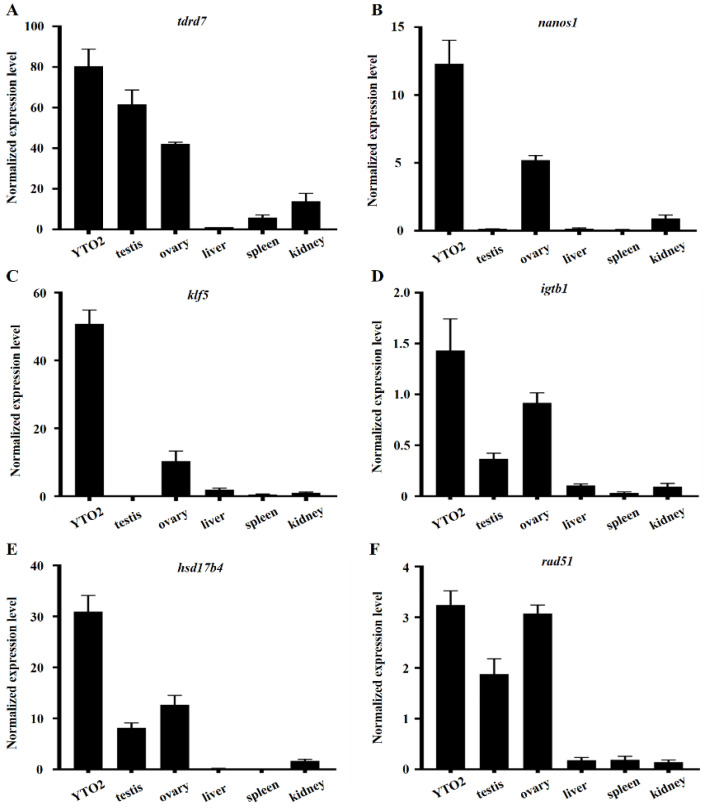
Expression level of germline marker genes in YTO2 cells and related tissues.

To assess the reliability of the RNA-seqs data, we further determined the expression profiles of germline marker genes *tdrd7*, *nanos1*, *klf5*, *igtb1*, *hsd17b4* and *rad51* in a panel of tissues or cells, including testis, ovary, the cultured ovarian cells, liver, spleen and kidney, since these genes have been documented in many species [37,38,39,40,41,42]. As described above, the morphology of cells became more uniform from passage 20 (Figure 1C), therefore, the expression profile of these marker genes was examined using cells at passage 20. QRT-PCR showed that *tdrd7* transcripts were highest expressed in YTO2 cells, richly in the testis and ovary, while weakly in somatic tissue liver, spleen and kidney (Figure 4A). *Nanos1* and *klf5* were expressed higher in YTO2 cells and ovary, and weakly in testis and somatic tissues (Figure 4B,C). *Igtb1*, *hsd17b4* and *rad51* were richly expressed in YTO2 cells and ovary, moderately in testis but expressed slightly in somatic tissues examined in this study (Figure 4D–F).

### 3.4. Immunofluorescence Identification of Germ Cell-Specific and Stem Cell-Specific Markers in the YTO2 Cells

**Figure 5:**
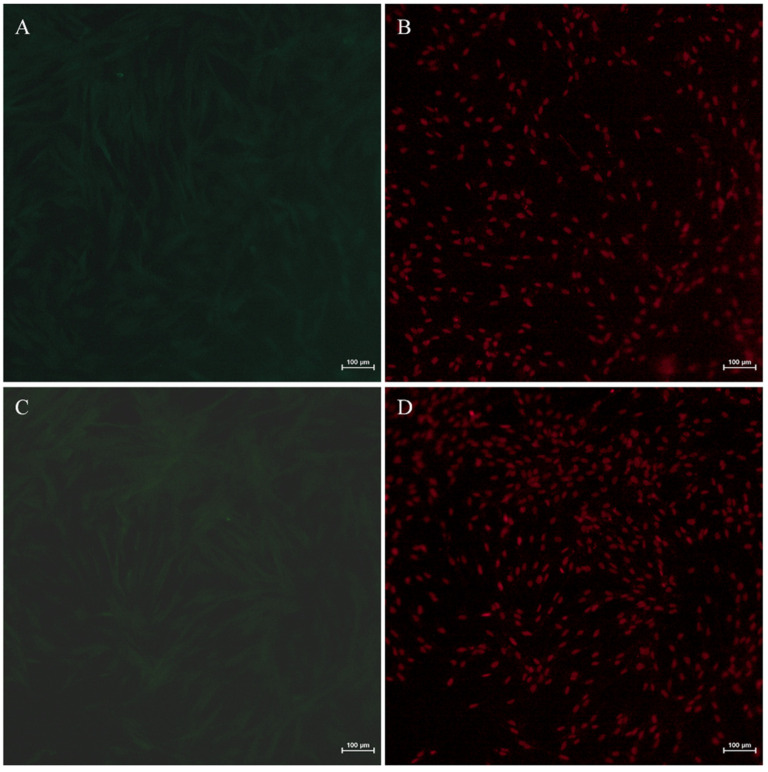
The YTO2 Cells immunostained with pre-immune serum as a negative control. The signals were visualized by TSA green staining (green) (**A**,**C**) and nuclei were stained red by propidium iodide (PI) (**B**,**D**). No signal was observed in YTO2 cells immunostained with both mouse serum (**A**,**B**) and rabbit serum (**C**,**D**).

**Figure 6:**
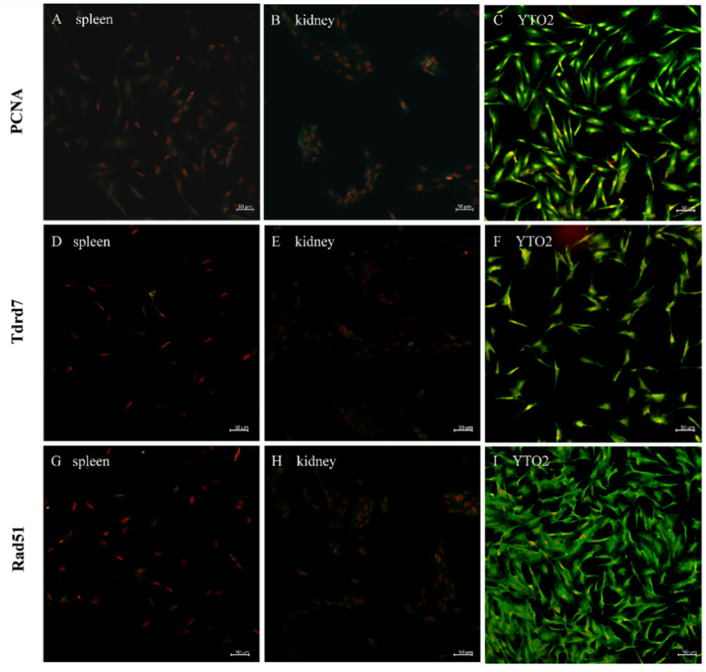
Fluorescence immunostaining of related proteins in YTO2 and somatic cells (**A**–**C**), PCNA; (**D**–**F**), Tdrd7 and (**G**–**I**), Rad51 and cultured somatic cells such as spleen cells and kidney cells. The signals were visualized by TSA green staining (green) and nuclei were counterstained with propidium iodide (PI; red).

In order to confirm the molecular characteristics ofYTO2 cells, the expression profile of PCNA, Tdrd7 and Rad51 proteins were examined using immunofluorescence staining. We first analyzed the immunohistochemical of YTO2 with mouse serum and rabbit serum as a negative control experiment. As shown in Figure 5, no signal was observed in YTO2 cells immunostained with both mouse serum (Figure 5A,B) and rabbit serum (Figure 5C,D). Moreover, to confirm the specificity of the antibodies used in this study, we conducted immunohistochemical analysis of PCNA, Tdrd7 and Rad51 in other cultured somatic cells including spleen cells and kidney cells as negative control Immunofluorescence signals of the proliferation marker, PCNA, were concentrated in the nuclei of the YTO2 cells but undetectable in spleen and kidney cells (Figure 6A–C). The germ cell-specific proteins, both Tdrd7 and Rad51, were abundantly observed in the cytoplasm with stronger signals in the cytoplasm of perinuclear region but absent in somatic cells (Figure 6D–I). Taken together, these results suggest that the isolated cells from the Asian yellow pond turtle ovary have the obvious characteristics of germline stem-like cells and called as female germ stem-like cells (fGSCs).

### 3.5. Meiosis Initiated by RA Induction

**Figure 7:**
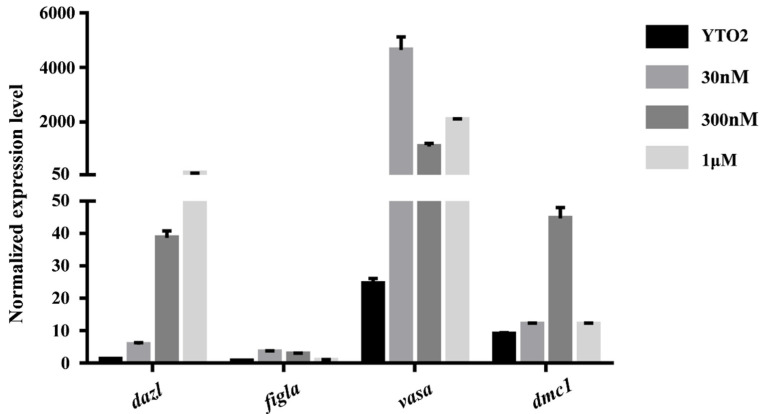
The alteration of marker genes expression in the YTO2 cells induced by retinoic acid (RA). The cultured YTO2 cells were exposed to RA (at the concentration of 30 nM, 300 nM and 1 µM) for 24 h, and the total RNA of treated and control YTO2 cells were extracted for qRT-PCR analysis. The result showed that the *dazl* and *vasa* mRNA expression were increased after RA treatment at all three dosage, *figla* was weakly expressed in control and 1 µM RA treated cells, but strongly in 30 nM and 300 nM RA treated cells. *Dmc1* was weakly expressed in control, 30 nM, 1 µM and strong in RA treated cells at the concentration of 300 nM.

To determine whether the cultured cells could respond to RA exposure or not, the related genes’ expression profiles were examined in YTO2 cells treated with RA (concentration of 30 nM, 300 nM and 1 µM). As shown in Figure 7, the germ cell marker *dazl* and *vasa* were significantly increased after RA treatment at all three dosage, and meiotic marker *figla* mRNA level displayed a slight elevation in YTO2 cells after RA exposure at 30 nM and 300 nM. The *dmc1* mRNA level was strongly increased after RA treatment at 300 nM. Moreover, we added control experiment to check possible effect of DMSO (the solvent control for RA) in the cell cultures (Figure S1). The result showed that two germ cell markers, *dazl* and *vasa* were highly expressed in YTO2 cells. Meiotic marker, *figla* was moderate in YTO2 cells, while *dmc1* was weakly expressed in YTO2 cells, suggesting that the amount of DMSO with the highest RA concentration did not affect the expression of these genes. These results suggested that RA can induce the expression of meiotic markers, as well as the initiation of proliferation and meiosis in YTO cells, the fGSCs, isolated from the Asian yellow pond turtle ovary.

### 3.6. Infection with HBAD-tdrd7-EGFP Adenovirus

**Figure 8:**
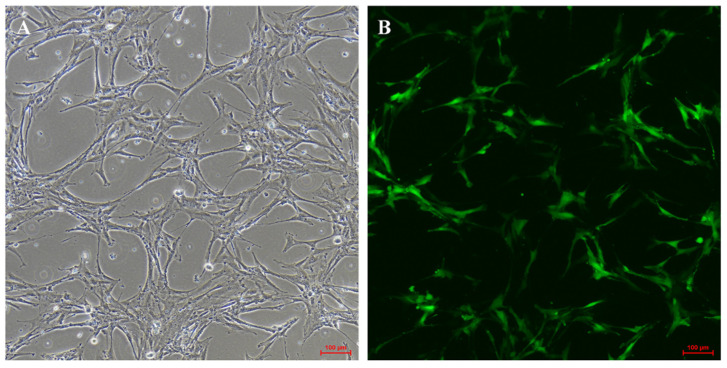
The YTO2 Cells transfected with pHBAd-BHG-EGFP adenovirus. At approximately 72 h after viruse infection, the EGFP expression was observed and taken images. (**A**) Bright field of transfected cells, 100 μm. (**B**). Fluorescent (green) image of pHBAd-BHG-EGFP infected cells. Scale bar, 100 μm.

In order to determine whether the YTO2 cells can be used as an effective system for genetic modification and investigations on the molecular mechanisms involved in reproductive and developmental process, such as oogenesis, germ cell differentiation, the transfection efficiency of adenovirus in YTO2 cells was evaluated via infecting the cells with HBAD-*tdrd7*-EGFP adenovirus. As shown in Figure 8, the EGFP can be observed in most of cells after 72 h transfection and the EGFP signals were mainly detected in the cytoplasm of cells indicating that the YTO2 cells were efficiently transfected with the adenovirus and properly expressed the exogenous genes.

## 4. Discussion

### 4.1. The Characteristics of YTO2 cells

Ovarian germline stem cells in adult mammalian ovary have been documented to provide some novel knowledge about ovarian regeneration, the delay of menopause, and therapy of ovarian dysfunction [43]. Though ovarian cells have been characterized in a wide mammalian tax including human [44], mice [5] and farm animals [9,45], but much less in aquatic species. Even in model fish species such as medaka [22] and zebrafish [46], the investigations were focused on the isolation of spermatogonial stem cells. Therefore, in this study, we successfully developed a rapid and efficient strategy for ovarian cells isolation and established a cell model for reproductive assays in the Asian yellow pond turtle. The ovarian cells, designed as YTO2, have been cultured and in vitro propagated more than 30 passages. After cryopreservation and thawing, the ovarian cells were successfully recovered for in vitro culture (Figure 1). Similar to the stem cells reported in other species, such as medaka [47], duck [45], and mammals [48], the morphology of YTO2 cells was predominant with fibroblast- and epithelial-like populations. After several passages of subculture, the round and polygonal cells appeared with little cytoplasm, large nuclei and prominent nucleoli, then the cells proliferated rapidly and formed the dominant population in the cultured cells (Figure 1).

Moreover, in terms of cell size, the diameter of ovarian stem cells varies among species. For example, the identified two populations of putative stem cells in adult mammals were comprised of small embryonic-like putative stem cells with 1–3 μm diameter and bigger progenitor stem cells with 4–7 μm diameter [7,49,50]. The ovarian stem cells in Chinese soft-shell turtle reached a diameter of approximately 10 μm [16]. In this study, the cell size of YTO2 was about 8–10 μm in diameter (Figure 1) which was similar to that of the Chinese soft-shell turtle germline stem cells and the bigger progenitor cells as previously reported [16].

Collectively, the morphological analysis suggests that cultured YTO2 cells possess the characteristics of germline stem-like cells and share high similarity with the PSO1 cells from the same order turtle [16]. This indicated that the culture conditions adopted in our study may be suitable for the in vitro culture and propagation of turtles’ female stem-like cells.

### 4.2. The Germline Properties of YTO2 Cells

To further define the origin of YTO2 cells, the cultured cells were subsequently characterized through profiling the expression of cell-specific markers. The transcriptome analysis of YTO2 detected a set of germline genes, cell pluripotency genes and somatic cell genes such as *nanos1*, *tdrd7*, *vasa*, *dnd1*, *dmc1*, *pou5f3*, *nanog*, *dmrt1* and *foxl2* (Figure 3). And, we also validated the expression profile of several selected markers in the YTO2 cells by qRT-PCR analysis including *tdrd7*, *nanos1*, *klf5*, *igtb1*, *hsd17b4* and *rad51* (Figure 4). Intriguingly, the RA induction could significantly increase the transcription level of late stages germ cell marker *vasa*, and meiotic markers, including *figla*, and *dmc1* genes while reduce the mRNA expression of early stage of germ cell marker *dazl*. These findings demonstrated that the YTO2 cells maybe is RA-signaling-responsive FGCs and could be induced into meiotic germ cells by RA treatment. And then the YTO2 cells could be considered as female germline stem-like cells (fGSCs) with meiotic potency.

Additionally, PCNA protein, a well-studied molecular marker related to cells’ self-renewal and/or mitotic activity [51] was examined and found to be highly expressed in the nuclei indicating a high potency of proliferation or mitotic activity of the YTO2 cells (Figure 5 and Figure 6). Likewise, the early germline markers, Tdrd7 and Rad51, majorly involved in primordial germ-cell formation as described previously [52,53] were dominantly detected in the perinuclear region cytoplasm of the newly isolated YTO2 cells (Figure 6). These proved that the YTO2 possese tha characteristics of germ cells at early stage.

### 4.3. The Potential Applications of YTO2 Cells

The applications of fGSCs are numerous, not only being applied in investigations on cell biology, but also in the field of clinical therapy. Likewise, fGSCs could provide a valuable tool for studying the mechanisms behind germ cell development and oogenesis in animals [54,55]. Moreover, appropriate genetic manipulations of the fGSCs have long been a major way to address the issues involved in oogenesis. As documented previously, fGSCs have also been genetically manipulated to produce transgenic mice using recombinant viruses containing vectors for different genes which exhibited an excellent tool for reproductive biologists [56]. In this study, we evaluated the feasibility of genetic manipulation in the YTO2 cells by infecting the YTO2 cells with HBAD-*tdrd7*-EGFP adenovirus. The EGFP was highly expressed in the cultured cells, indicating that the adenovirus can effectively infect the YTO2 cells and the expression of exogenous genes could be effectively driven in the Asian yellow pond turtle ovarian cell line (Figure 8). Since most turtle species have a long period of sexual maturation, if the young individuals harvested before they become sexual maturity, they have no chance to breed the next generation and expand the population [57]. Moreover, turtle is an egg-laying breeding animal with hard shell, which makes it difficult for gene manipulation. And, so far, CRISPR/Cas9-mediated gene editing technology has limitedly been applied in turtle. Therefore, YTO2 cells with a high efficiency of virus infection would make it a valuable tool for genetic modfication in turtles. It is of great significance for analyzing the gene’ functions and investigating the molecular networks involved in germ cell development, sexual differentiation and other reproduction process in the Asian yellow pond turtle.

On the other hand, the significant application of YTO2 cells is also embodied in the potential contributions in the field of fertility preservation and long-term germ cell cryopreservation of threatened species. It has been reported that the cryopreserved fGSCs can be transplanted into a woman’s ovaries and form new follicles, or be cultured in vitro and differentiate into the mature oocyte stage in the ovarian cortex, which can eventually be used for in vitro fertilization [10]. In terms of aquatic animals, the cryopreserved fish ovary tissues was proven to be capable of giving rise to offspring through germ cell transplantation such as medaka [58], rainbow trout [55] and zebrafish [46]. Therefore, it is conceivable that fGSCs could be a useful fertility preservation strategy to intervene and improve ovarian function.

## 5. Conclusions

In this study, we isolated an ovarian cell line (fGSCs), from the juvenile Asian yellow pond turtle in vitro. Our study aims to provide a valuable tool to elaborate the molecular mechanisms behind germ cells development, differentiation and oogenesis in the turtle even in reptiles, and also, have potential applications to enrich the conservation strategy of aquatic animal germplasm resources.

## Figures and Tables

**Table 1:** The primer pairs used for RT-PCR in this study.

Gene Name	Primer Sequence	Amplicon Size (bp)
*β-actin*	F: 5′-GTGGCTATCCAGGCTGTGCT-3′ R: 5′-TGGTGGTGAAGCTGTAGCCTC-3′	168
*tdrd7*	F: 5′-ACCTCCTCTACCCTTACCGA-3′ R: 5′-TGATGCCTCTTCTGACCACT-3′	283
*rad51*	F: 5′-AAAGTGATTCCAATGGGTTT-3′ R: 5′-AGCTGGGTCTGATGGTCTGT-3′	371
*nanos1*	F: 5′-GCGCTGCACTTGCACGACTT-3′ R: 5′-GGGCGACGGCTTCTTTGTTATT-3′	283
*igtb1*	F: 5′-CGACTTCTGCCCGATGTAAT-3′ R: 5′-TGGCTGGATCTGGGTAATGT-3′	173
*hsd17b4*	F: 5′- GTAACAGTGAACCCGCCTAA-3′ R: 5′- TTTCCCTCCTTCCACATCTC-3′	308
*klf5*	F: 5′- TACCCACATCAAGACAGAACC-3′	261
	R: 5′-GCTGCCTGAGCAGTAGAATT-3′	

## Data Availability

Transcriptome raw data of cultured ovarian cells of *Mauremys mutica* are deposited at NCBI Sequence Read Archive database under the BioSample accession number of SRR18498473 (BioProject ID: PRJNA820525) (Title: Characterization of the in vitro cultured ovarian cells in *Mauremys mutica*. Available online: https://www.ncbi.nlm.nih.gov/bioproject/PRJNA820525 (accessed on 28 March 2022).

## Supplementary Materials

The following supporting information can be downloaded at: https://www.mdpi.com/article/10.3390/biology11101404/s1. Figure S1. The alteration of marker genes expression in the YTO2 cells induced by Dimethyl sulfoxide (DMSO). The cultured YTO2 cells were exposed to RA (at the concentration of 30 nM, 300 nM and 1 µM) for 24 h, and the total RNA of treated and control YTO2 cells were extracted for PCR analysis. The result showed that two germ cell markers, dazl and vasa were highly expressed in YTO2 cells. Meiotic marker, figla was moderate in YTO2 cells, while dmc1 was weakly expressed in YTO2 cells; Table S1. Summary statistics of sequencing data; Table S2. Detail of the genes identified in YTO2 using RNA-seq; Table S3. Detail of the genes discussed in the text.
