# Supplementary material for: Characterization of the In Vitro Cultured Ovarian Cells in the Asian Yellow Pond Turtle (Mauremys mutica)
Source: Biology (Basel). 2022 Sep 26;11(10):1404. doi: 10.3390/biology11101404 (PMC9598145; doi:10.3390/biology11101404)
Supplement: Supplementary file 1 [file biology-11-01404-s001.zip › supplementary file0722.pdf]

Characterization of the *in vitro* cultured ovarian cells in the Asian yellow pond turtle (*Mauremys mutica*)

Xiaoli Liu[1], Fang Liu[1], Haoyang Xu[1], Yanping Yang[1], Yakun Wang[1], Xiaoyou Hong[1], Wei Li[1], Lingyun Yu[1], Chen Chen[1], Hongyan Xu[1,2]*, Xinping Zhu[1]*

[1] Key Laboratory of Tropical and Subtropical Fishery Resources Application and Cultivation, Ministry of Agriculture and Rural Affairs, Pearl River Fisheries Research Institute, Chinese Academy of Fishery Sciences, Guangzhou, China, 510380

[2] Key Laboratory of Freshwater Fish Reproduction and Development (Ministry of Education), College of Fisheries, Southwest University, Chongqing, China, 402460

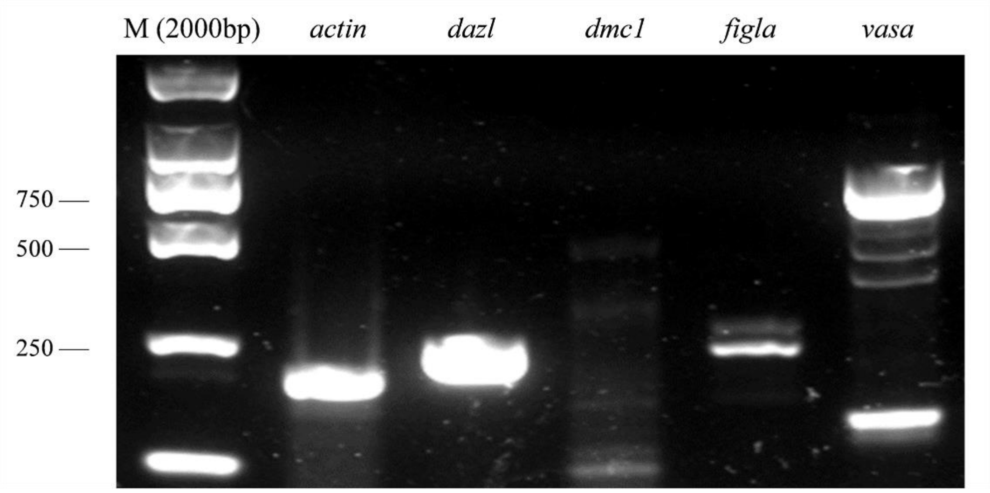

Figure S1. The alteration of marker genes expression in the YTO2 cells induced by Dimethyl sulfoxide (DMSO). The cultured YTO2 cells were exposed to RA (at the concentration of 30 nM, 300 nM and 1 μM) for 24 hours, and the total RNA of treated and control YTO2 cells were extracted for PCR analysis. The result showed that two germ cell markers, *dazl* and *vasa* were highly expressed in YTO2 cells. Meiotic marker, *figla* was moderate in YTO2 cells, while *dmc1* was weakly expressed in YTO2 cells.

Table S1. Summary statistics of sequencing data

**Sample**	Raw Reads	Clean Reads	Total mapped reads	Multiple mapped	Uniquely mapped	Q30	GC
YTO2-1	51.63 M	50.27 M	47,466，940(94.42%)	2,158,288(4.29%)	45,308,652(90.12%)	94.40%	50.34%
YTO2-2	57.68 M	55.93 M	51,743,669(92.52%)	2,308,517(4.13%)	49,435,152(88.39%)	93.85%	50.04%
Average	54.66 M	53.1M	49,605,305(93.47%)	2,233,402(4.21%)	47,371,902(89.26%)	94.13%	50.19%

Note: ″M″: million

Table S3. Detail of the genes discussed in the text.

	gene symbol	YTO2_1	YTO2_1	description
germline genes	*ddx4*	0.9989007	0.5831704	probable ATP-dependent RNA helicase DDX4 isoform X1 [*Chelonoidis abingdonii*]
	dazl	0	0.0479189	deleted in azoospermia like
	dnd1	0.1647492	0.033431	dead end protein homolog 1 isoform X1 [*Chelonoidis abingdonii*]
	piwil1	0.0302911	0	piwi-like protein 1 isoform X1 [*Chelonoidis abingdonii*]
	piwil2	0.4835382	0.5066843	N-acetylglucosamine-1-phosphodiester alpha-N-acetylglucosaminidase [*Platysternon megacephalum*]
	mre11	5.2246185	4.9816236	double-strand break repair protein MRE11 [*Gopherus evgoodei*]
	rad50	5.3908906	4.7497469	putative E3 ubiquitin-protein ligase UNKL [*Platysternon megacephalum*]
	dmc1	0.038648	0.1764557	DNA meiotic recombinase 1 [*Columba livia*]
	nanos1	49.407998	47.05324	nanos homolog 1 [*Gopherus evgoodei*]
	msh2	15.116453	15.79862	DNA mismatch repair protein Msh2 isoform X1 [*Chelonoidis abingdonii*]
	sycp3	0.818219	0.9735595	synaptonemal complex protein 3 isoform X2 [*Pelodiscus sinensis*]
	figla	0.0484598	0	factor in the germline alpha [*Gopherus evgoodei*]
	slbp2	14.051173	15.336126	histone RNA hairpin-binding protein isoform X2 [*Terrapene carolina triunguis*]
	sycp3	0.818219	0.9735595	synaptonemal complex protein 3 isoform X2 [*Pelodiscus sinensis*]
	dmrt2	0.5492919	0.6611758	glutathione peroxidase 3 [*Platysternon megacephalum*]
	tdrd7	6.5820831	5.9375251	tudor domain-containing protein 7 isoform X1 [*Chelonoidis abingdonii*]
	nr5a1	1.7878892	1.9545177	steroidogenic factor-1 [*Mauremys reevesii*]
	fancd2	1.4835573	1.1372781	E3 ubiquitin-protein ligase makorin-1 [*Platysternon megacephalum*]
	gdnf	5.635788	5.2670267	glial cell line-derived neurotrophic factor isoform X1 [*Pelodiscus sinensis*]
	rad51	7.5428651	7.5601774	DNA repair protein RAD51 homolog 1 isoform X1 [*Gopherus evgoodei*]
	ybx2	0.4647819	0.9902947	Y-box-binding protein 2 [*Chelonoidis abingdonii*]

slbp2	14.051173	15.336126	histone RNA hairpin-binding protein isoform X2 [*Terrapene carolina triunguis*]
CD73	0.924619	1.3923687	piezo-type mechanosensitive ion channel component 2 [*Platysternon megacephalum*]
CD90	14.143935	14.089554	thy-1 membrane glycoprotein [*Gopherus evgoodei*]
CD105	39.570159	44.321225	endoglin [*Gopherus evgoodei*]
CD61	123.3671	110.73413	integrin beta-3 isoform X1 [*Chelonoidis abingdonii*]
itgb1	647.04905	601.6362	integrin beta-1 isoform X2 [*Terrapene carolina triunguis*]
itgb6	0.572165	0.272592	integrin beta-6 isoform X1 [*Gopherus evgoodei*]
ccnb1	14.683519	16.874192	G2/mitotic-specific cyclin-B1 [*Gopherus evgoodei*]
ccna2	4.7820979	4.3544695	cyclin-A2 [*Terrapene carolina triunguis*]
cdc20	7.2003414	7.1340291	cell division cycle protein 20 homolog isoform X1 [*Chelonoidis abingdonii*]
plk3	9.9738124	8.9912434	serine/threonine-protein kinase PLK3 [*Gopherus evgoodei*]
PCNA	61.554016	63.951863	proliferating cell nuclear antigen [*Chrysemys picta bellii*]
mcm4	11.339022	12.343582	DNA replication licensing factor MCM4 isoform X1 [*Chelonoidis abingdonii*]
kif22	0	0.0397332	kinesin-like protein KIF22 [*Chelonoidis abingdonii*]
pcbp2	154.21063	181.24115	poly(rC)-binding protein 2 isoform X1 [*Chrysemys picta bellii*]
cdk1	19.904881	21.141359	V-type proton ATPase subunit H [*Platysternon megacephalum*]
siva1	70.218628	95.768722	apoptosis regulatory protein Siva [*Gopherus evgoodei*]
prmt1	132.51878	146.87333	ankyrin repeat and SOCS box protein 6 [*Platysternon megacephalum*]
bub3	38.874586	41.352736	hypothetical protein [*Bos mutus*]
cdc26	30.183873	30.669669	anaphase-promoting complex subunit CDC26 [*Chelonoidis abingdonii*]
ccnb3	6.0731504	6.6491214	G2/mitotic-specific cyclin-B3 isoform X1 [*Gopherus evgoodei*]
vdac2	103.51996	113.82122	voltage-dependent anion-selective channel protein 2 [*Chrysemys picta bellii*]
rbx1	72.067248	72.661155	E3 ubiquitin-protein ligase RBX1 [*Chrysemys picta bellii*]
mcm7	18.724784	20.915789	DNA replication licensing factor MCM7 [*Aquila chrysaetos chrysaetos*]
orc3	2.8809614	2.9410185	origin recognition complex subunit 3 isoform X1 [*Chrysemys picta bellii*]

	plk1	10.011971	13.180602	long-chain-fatty-acid--CoA ligase 5 [*Platysternon megacephalum*]
	sod2	79.734609	99.011703	mitochondrial manganese superoxide dismutase [*Mauremys reevesii*]
	skp1	213.20533	217.14728	hypothetical protein [*Chiloscyllium punctatum*]
	hsd17b4	29.329903	28.233718	peroxisomal multifunctional enzyme type 2 isoform X1 [*Gopherus evgoodei*]
	hacd4	4.3065705	4.9599243	very-long-chain (3R)-3-hydroxyacyl-CoA dehydratase 1 isoform X1 [*Chelonoidis abingdonii*]
	hacd1	31.329618	33.640823	histone deacetylase 1 [*Serinus canaria*]
	acaa1	24.13113	23.833942	tudor domain-containing protein 3 [*Platysternon megacephalum*]
somatic cell genes	*foxl2*	0.1823301	0.1664933	forkhead box protein L2-like [*Chelonoidis abingdonii*]
	dmrt1	0.135677	0.0743354	doublesex- and mab-3 related transcription factor 1 [*Mauremys reevesii*]
	amh	0.1437601	0.0492275	muellerian-inhibiting factor [*Chelonoidis abingdonii*]
	sox9	0.2062153	0.437351	transcription factor SOX-9 [*Terrapene carolina triunguis*]
	sox8	1.5522183	2.0818006	transcription factor SOX-8 [*Gopherus evgoodei*]
	fgf9	0.1754756	0.0300439	fibroblast growth factor 9 [*Taeniopygia guttata*]
	zfpm2	3.8079592	3.6224704	zinc finger protein ZFPM2 isoform X1 [*Terrapene carolina triunguis*]
	map3k4	4.2931003	4.2351543	mitogen-activated protein kinase kinase kinase 4 isoform X3 [*Chelonoidis abingdonii*]
	fst	124.24083	117.7703	focadhesin [*Platysternon megacephalum*]
	rspo1	0.5257559	0.6837644	R-spondin-1 isoform X2 [*Chrysemys picta bellii*]
	wnt4	0.0214522	0	protein Wnt-4 isoform X1 [*Chrysemys picta bellii*]
	spa17	3.5580618	4.6328568	sperm surface protein Sp17 [*Chelonoidis abingdonii*]
	hsd17b2	0.9849342	0.5231117	glyoxylate reductase/hydroxypyruvate reductase-like [*Platysternon megacephalum*]
	hsd17b1	4.2491867	5.5861127	estradiol 17-beta-dehydrogenase 1 [*Chelonia mydas*]
	spata7	2.1791562	2.2913765	spermatogenesis-associated protein 7 isoform X1 [*Chelonoidis abingdonii*]
	cyp11b	0.7464259	0.6389934	cytochrome P450 11B, mitochondrial-like [*Gopherus evgoodei*]
	srd5a1	1.0513777	1.2160727	3-oxo-5-alpha-steroid 4-dehydrogenase 1 isoform X1 [*Chrysemys picta bellii*]
	h2a	1.5864369	2.0349979	histone h2a [*Lynx pardinus*]

	sox2	0.6942051	0.6909597	transcription factor SOX-2 [*Platysternon megacephalum*]
	pou5f3	1.785611	1.1401359	POU domain, class 5, transcription factor 3 isoform X1 [*Gopherus evgoodei*]
	klf5	8.50921	7.6180349	Krueppel-like factor 5 [*Gopherus evgoodei*]
	nanog	0	0.0195826	homeobox protein NANOG-like [*Chrysemys picta bellii*]
	tcl1	0.0766445	0.104981	protein p13 MTCP-1-like [*Terrapene carolina triunguis*]
	esrrb	0.0349382	0.0063807	steroid hormone receptor ERR2 [*Pogona vitticeps*]
	sall4	4.2018325	3.0057385	sal-like protein 4 [*Terrapene carolina triunguis*]
	tcf7l1	18.121512	17.817572	transcription factor 7-like 1 isoform X1 [*Terrapene carolina triunguis*]
cell pluripotency genes	*mycn*	0	0.0469822	N-myc proto-oncogene protein-like [*Chelonoidis abingdonii*]
	fbxo15	0	0.014479	F-box only protein 15 isoform X1 [*Chrysemys picta bellii*]
	zfx	8.8903313	10.045974	zinc finger X-chromosomal protein [*Chelonoidis abingdonii*]
	FoxD3	0	0.0173177	LOW QUALITY PROTEIN: forkhead box protein D3 [*Chrysemys picta bellii*]
	hmga2	3.3744901	3.0543597	high mobility group protein HMGI-C isoform X1 [*Pelodiscus sinensis*]
	nr6a1	0.1653544	0.1342152	nuclear receptor subfamily 6 group A member 1 isoform X4 [*Chrysemys picta bellii*]
	stat3	18.482631	16.937332	signal transducer and activator of transcription 3 isoform X1 [*Chelonia mydas*]
	lef1	1.1641186	1.6724626	lymphoid enhancer-binding factor 1 isoform X2 [*Chelonoidis abingdonii*]
	tcf7	3.0303746	2.7500818	transcription factor 7 isoform X1 [*Gopherus evgoodei*]
	tcf7l1	18.121512	17.817572	transcription factor 7-like 1 isoform X1 [*Terrapene carolina triunguis*]
	tcf7l2	5.1539342	6.1445973	transcription factor 7-like 2 isoform X15 [*Chrysemys picta bellii*]
